# Plasma Big Endothelin-1 Levels and Long-Term Outcomes in Patients With Atrial Fibrillation and Acute Coronary Syndrome or Undergoing Percutaneous Coronary Intervention

**DOI:** 10.3389/fcvm.2022.756082

**Published:** 2022-03-03

**Authors:** Si-qi Lyu, Jun Zhu, Juan Wang, Shuang Wu, Han Zhang, Xing-hui Shao, Yan-min Yang

**Affiliations:** Emergency and Critical Care Center, State Key Laboratory of Cardiovascular Disease, National Center for Cardiovascular Diseases, Fuwai Hospital, Chinese Academy of Medical Sciences and Peking Union Medical College, Beijing, China

**Keywords:** plasma big ET-1 levels, atrial fibrillation, acute coronary syndrome, percutaneous coronary intervention, all-cause mortality, net adverse clinical events

## Abstract

**Background:**

This study aimed to evaluate the association between plasma big ET-1 levels and long-term outcomes in patients with atrial fibrillation (AF) and acute coronary syndrome (ACS) or undergoing percutaneous coronary intervention (PCI).

**Methods:**

A total of 930 patients were enrolled and followed up for a median duration of 2.3 years. According to the optimal cutoff of big ET-1 for predicting all-cause death, these patients were divided into two groups. The primary endpoints were all-cause death and net adverse clinical events (NACE). The secondary endpoints included cardiovascular death, major adverse cardiovascular events (MACE), BARC class ≥ 3 bleeding, and BARC class ≥ 2 bleeding. Cox regressions were performed to evaluate the association between big ET-1 and outcomes.

**Results:**

Based on the optimal cutoff of 0.54 pmol/l, 309 patients (33.2%) had high big ET-1 levels at baseline. Compared to the low big ET-1 group, patients in the high big ET-1 group tended to have more comorbidities, impaired cardiac function, elevated inflammatory levels, and worse prognosis. Univariable and multivariable Cox regressions indicated that big ET-1 ≥ 0.54 pmol/l was associated with increased incidences of all-cause death [HR (95%CI):1.73 (1.10–2.71), *p* = 0.018], NACE [HR (95%CI):1.63 (1.23–2.16), *p* = 0.001], cardiovascular death [HR (95%CI):1.72 (1.01–2.92), *p* = 0.046], MACE [HR (95%CI):1.60 (1.19–2.16), *p* = 0.002], BARC class ≥ 3 [HR (95%CI):2.21 (1.16–4.22), *p* = 0.016], and BARC class ≥ 2 bleeding [HR (95%CI):1.91 (1.36–2.70), *p* < 0.001]. Subgroup analysis indicated consistent relationships between the big ET-1 ≥ 0.54 pmol/l and the primary endpoints.

**Conclusion:**

Elevated plasma big ET-1 levels were independently associated with increased risk of all-cause death, NACE, cardiovascular death, MACE, BARC class ≥ 3 bleeding, and BARC class ≥ 2 bleeding in patients with AF and ACS or undergoing PCI.

## Introduction

Due to abundant common risk factors, the coexistence of coronary heart disease (CAD) and atrial fibrillation (AF) is prevalent, resulting in worse prognoses and increased healthcare burdens (1, 2). Approximately 6–21% of patients with CAD are complicated with AF (3), while CAD is estimated to occur in 20–30% of patients with AF (4–6). It's well-established that inflammation, endothelial dysfunction, atrial and ventricular remodeling play important roles in the occurrence and progression of CAD and AF. Endothelin-1 (ET-1), a peptide derived from endothelial cells, has been indicated to be associated with endothelial dysfunction and inflammation (7, 8). Meanwhile, as an autocrine and paracrine mediator, ET-1 is associated with myocardial remodeling involving cardiac hypertrophy, dilatation, and fibrosis (9–11).

Big ET-1 is the precursor of ET-1 with no biological function but a longer half-life in the peripheral circulation. In clinical settings, big ET-1 is more easily measured and widely used to evaluate the activity of the endothelial system (7, 8). Several studies have demonstrated that elevated big ET-1 is a risk factor for adverse outcomes in patients with heart failure (12), CAD (13–16), AF (17), and hypertrophic cardiomyopathy (18). However, the association between plasma big ET-1 levels and long-term outcomes in patients with AF and acute coronary syndrome (ACS) or undergoing percutaneous coronary intervention (PCI) has not been evaluated before. Therefore, we conducted a *post-hoc* analysis of a cohort study in Chinese patients with AF and ACS or undergoing PCI to explore this issue.

## Methods

### Study Population

This study enrolled consecutive patients with AF and ACS or undergoing PCI in Fuwai Hospital. Inclusion criteria included: (1) patients aged ≥18 years. (2) patients had paroxysmal, persistent, or permanent AF confirmed by clinical records and electrocardiographic evidence (including electrocardiograms, Holter, and rhythm strips). (3) patients were diagnosed with ACS (unstable angina, non-ST-segment elevated myocardial infarction, or ST-segment elevated myocardial infarction), or underwent PCI during hospitalization. Exclusion criteria were as follows: patient's refusal to participate, coagulopathy or thrombocytopenia (platelet count <50^*^10^9^/L), contraindications to anticoagulant or antiplatelet agents, and life expectancy <12 months. The study was approved by the ethics committee of Fuwai Hospital and obeyed the Declaration of Helsinki. Informed consent for participation was provided by each patient. Patients were treated with anticoagulant and antiplatelet therapy according to clinical guidelines.

### Baseline Information

Patient's baseline information was obtained by interviewing the patients, consulting their physicians, and reviewing medical records. Based on the above data, the CHA_2_DS_2_-VASc score and the HAS-BLED score were calculated for each patient according to their definitions. BMI was calculated by dividing weight in kilograms by the square of height in meters. Creatinine clearance was calculated according to the Cockcroft-Gault formula. Creatinine clearance <60 ml/min was defined as renal insufficiency.

### Laboratory Tests of Plasma Big ET-1 Levels

On admission, venous blood samples were drawn from all patients and collected into ethylene diamine tetraacetic acid (EDTA)-treated tubes according to venous blood specimen collection standards. The concentration of big ET-1 was measured using a highly sensitive and specific commercial sandwich enzyme immunoassay (BI-2008 2H, Biomedica, Wien, Austria).

### Follow-Up and Outcomes

Outcome data were obtained by trained research personnel via telephone interview, outpatient visit, or delivery of medical records. Follow-up was completed by April 2021, with a median duration of 2.3 years. The primary endpoints were all-cause death and net adverse clinical events (NACE, referring to a composite of all-cause death, stroke, non-central nervous system embolism, myocardial infarction, definite or probable stent thrombosis, target vessel revascularization, and major bleeding). The secondary endpoints included cardiovascular death and major adverse cardiovascular events (MACE, a composite of all-cause death, stroke, non-central nervous system embolism, myocardial infarction, definite or probable stent thrombosis, and target vessel revascularization). The safety endpoints were defined according to the bleeding academic research consortium (BARC) criteria as major bleeding (BARC 3a, 3b, 3c, and 5) and any bleeding (BARC 2, 3a, 3b, 3c, and 5) (19). All outcomes were blindly adjudicated by an independent committee according to standardized principles. Death and the cause were ascertained by medical records obtained and reports of the physicians. Stroke referred to focal neurological deficits lasting more than 24 h and confirmed by imaging. Non-central nervous system embolism was defined as a vascular occlusion due to embolism confirmed by imaging or surgery. Myocardial infarction was defined based on the 4th Definition of Myocardial Infarction (20). Stent thrombosis was defined according to the Academic Research Consortium statement (21). Target vessel revascularization referred to any repeat PCI or coronary artery bypass grafting (CABG) of any segment of the target vessel (21).

### Statistical Analysis

Continuous variables are presented as median (interquartile range) and compared by Mann-Whitney U test for the data are not normally distributed. Categorical variables are presented as frequency (percentage) and compared by Pearson's χ2 test or Fisher's exact test. Receiver operating characteristic (ROC) curve analysis was performed to identify the optimal cutoff of plasma big ET-1 level with the highest Youden index (Sensitivity+Specificity-1). Patients were divided into a low big ET-1 level group and a high big ET-1 level group according to the cutoff value of plasma big ET-1 level. Logistic regression (backward likelihood ratio method) was utilized to identify factors related to high plasma big ET-1 levels. The Kaplan-Meier survival curves were constructed and log-rank tests were utilized to illustrate survival discrepancies between the two groups. Univariable and multivariable Cox proportional hazard regression were performed for all endpoints, in which hazard ratio (HR) and 95% confidence interval (CI) were calculated. To avoid overfitting, variables with a *p*-value <0.10 in the univariable analysis or clinically relevant with endpoints were entered into the multivariable regression models with the backward LR (likelihood ratio) method. Subgroup analyses were performed to assess the homogeneity of the association between plasma big ET-1 levels and all-cause mortality. A *p*-value of <0.05 was defined as statistically significant. All statistical analyses were performed by SPSS version 25.0 (IBM Corporation, New York, USA).

## Results

### Baseline Information

A total of 930 patients with AF and ACS or undergoing PCI were recruited from September 2016 to March 2020. Details of the subject selection process are displayed in Supplementary Figure 1. According to the ROC analysis, the c-statistic of plasma big ET-1 level for predicting all-cause mortality was 0.746, and the optimal cutoff value of plasma big ET-1 level was 0.54 pmol/l, with a sensitivity of 66.97% and a specificity of 71.25%. Based on this threshold, patients were divided into two groups. The baseline characteristics of included patients are summarized in Table 1. Among the 930 patients with a median age of 68 years, 680 patients (73.1%) were male and 309 patients (33.2%) had high big ET-1 levels (≥0.54 pmol/l). Compared to patients with big ET-1 <0.54 pmol/l, patients with big ET-1 ≥ 0.54 pmol/l were more likely to have elder ages, non-paroxysmal AF, diabetes mellitus, previous myocardial infarction, previous coronary stent implantation, previous CABG, heart failure, previous stroke/transient ischemic attack, peripheral arterial disease, previous bleeding events, creatinine clearance <60 ml/min, and chronic obstructive pulmonary disease (all *p* < 0.05), which corresponded with the relatively higher CHA_2_DS_2_-VASc score (*p* < 0.001) and HAS-BLED score (*p* < 0.001). Patients in the high big ET-1 group tended to have higher admission heart rates, white blood cell counts, neutrophil-lymphocyte ratios, red cell distribution widths, uric acid, free fatty acid, hemoglobin A1c, N-terminal pro-B type natriuretic peptide, high sensitivity C reactive protein, left atrial diameters, left ventricular end-diastolic diameters, but have lower admission systolic blood pressure, hemoglobin, platelet counts, creatinine clearance, low-density lipoprotein cholesterol, and left ventricular ejection fractions (all p <0.05). As to treatments, patients with big ET-1 ≥ 0.54 pmol/l had a lower rate of receiving triple antithrombotic therapy, but a higher rate of spironolactone and diuretic use (all *p* < 0.001).

**Table 1:** Baseline characteristics of patients with AF and ACS or undergoing PCI.

**Variables**	**Total (*n* = 930)**	**Big ET-1 <0.54 pmol/l (*n* = 621)**	**Big ET-1 ≥0.54 pmol/l (*n* = 309)**	***p*-value**
**Demographics**				
Age (years)	68 (62, 73)	67 (61, 72)	69 (63, 75)	0.002
Male gender, *n* (%)	680 (73.1)	456 (73.4)	224 (72.5)	0.822
Body mass index (kg/m2)	25.6 (23.6, 27.8)	25.7 (23.7, 27.8)	25.4 (23.3, 27.7)	0.065
Heart rate (bpm)	70 (66, 82)	70 (66, 79)	74 (66, 91)	<0.001
Systolic blood pressure (mmHg)	127 (118, 140)	128 (120, 140)	126 (114, 139)	0.030
Diastolic blood pressure (mmHg)	78 (70, 82)	78 (70, 82)	76 (69, 84)	0.227
Smoking history, *n* (%)	496 (53.3)	331 (53.3)	165 (53.4)	1.000
Drinking history, *n* (%)	389 (41.8)	266 (42.8)	123 (39.8)	0.417
**Type of AF**, ***n*** **(%)**				<0.001
Paroxysmal	417 (44.8)	304 (49.0)	113 (36.6)	
Persistent	218 (23.4)	129 (20.8)	89 (28.8)	
Permanent	295 (31.7)	188 (30.3)	107 (34.6)	
**Qualifying index event**, ***n*** **(%)**				<0.001
ACS undergoing PCI	339 (36.5)	243 (39.1)	96 (31.1)	
ACS without PCI this time	482 (51.8)	284 (45.7)	198 (64.1)	
Elective PCI	109 (11.7)	94 (15.1)	15 (4.9)	
**Medical histories**, ***n*** **(%)**				
Hypertension	736 (79.1)	499 (80.4)	237 (76.7)	0.228
Hyperlipidemia	532 (57.2)	346 (55.7)	186 (60.2)	0.219
Diabetes mellitus	442 (47.5)	280 (45.1)	162 (52.4)	0.041
Previous myocardial infarction	310 (33.3)	173 (27.9)	137 (44.3)	<0.001
Previous coronary stent implantation	254 (27.3)	152 (24.5)	102 (33.0)	0.008
Previous coronary artery bypass grafting	90 (9.7)	46 (7.4)	44 (14.2)	0.001
Heart failure	364 (39.1)	169 (27.2)	195 (63.1)	<0.001
Previous stroke/transient ischemic attack	226 (24.3)	137 (22.1)	89 (28.8)	0.030
Peripheral arterial disease	188 (20.2)	106 (17.1)	82 (26.5)	<0.001
Previous bleeding events	74 (8.0)	39 (6.3)	35 (11.3)	0.011
Creatinine clearance <60 ml/min	91 (9.8)	47 (7.6)	44 (14.2)	0.002
Chronic obstructive pulmonary disease	331 (35.6)	175 (28.2)	156 (50.5)	<0.001
CHA2DS2-VASc score	4 (3, 5)	3 (2, 5)	4 (3, 6)	<0.001
HAS-BLED score	3 (2, 4)	3 (2, 3)	3 (2, 4)	<0.001
**Laboratory tests and echocardiographic parameters, median (IQR)**				
White blood cell count (*10^9^/l)	6.7 (5.5, 8.2)	6.7 (5.5, 7.8)	6.9 (5.6, 8.8)	0.011
Neutrophil-lymphocyte ratio	2.7 (2.0, 4.0)	2.6 (1.9, 3.6)	3.2 (2.1, 5.0)	<0.001
Hemoglobin (g/l)	144.0 (132.0, 156.0)	147.0 (135.0, 157.0)	139.0 (123.0, 150.0)	<0.001
Red blood cell distribution width (%)	13.0 (12.5, 13.6)	12.9 (12.4, 13.3)	13.2 (12.7, 14.3)	<0.001
Platelet count (*10^9^/l)	204.0 (166.0, 243.5)	208.0 (174.0, 248.0)	194.0 (153.0, 235.0)	<0.001
Creatinine clearance (ml/min)	68.2 (53.3, 83.0)	71.9 (58.7, 85.9)	60.0 (45.0, 74.7)	<0.001
Uric acid (umol/l)	379.6 (305.4, 467.7)	358.4 (290.2, 438.9)	424.0 (340.0, 520.0)	<0.001
Free fatty acid (mmol/l)	0.6 (0.4, 0.8)	0.5 (0.4, 0.7)	0.6 (0.4, 0.8)	<0.001
Low-density lipoprotein cholesterol (mmol/l)	2.1 (1.7, 2.6)	2.1 (1.7, 2.7)	2.0 (1.6, 2.5)	0.002
Hemoglobin A1c (%)	6.4 (5.9, 7.4)	6.3 (5.9, 7.3)	6.6 (6.0, 7.7)	0.012
N-terminal pro-B type natriuretic peptide (pg/ml)	959.4 (375.4, 2445.0)	663.3 (272.5, 1445.0)	2234.0 (877.4, 5490.0)	<0.001
High sensitivity C reactive protein (mg/l)	2.1 (0.9, 6.1)	1.7 (0.8, 4.3)	3.9 (1.3, 10.1)	<0.001
Left atrial diameter (mm)	43 (40, 48)	43 (39, 47)	45 (41, 50)	<0.001
Left ventricular end-diastolic diameter (mm)	50 (46, 56)	49 (46, 54)	53 (48, 59)	<0.001
Left ventricular ejection fraction (%)	60 (48, 63)	60 (55, 64)	52 (39, 60)	<0.001
**Medications**, ***n*** **(%)**				
**Antiplatelet agents**				
Aspirin	546 (58.7)	389 (62.6)	157 (50.8)	<0.001
Clopidogrel	877 (94.3)	589 (94.8)	288 (93.2)	0.385
Ticagrelor	11 (1.2)	8 (1.3)	3 (1.0)	1.000
**Oral anticoagulants**				0.979
Warfarin	319 (34.3)	212 (34.1)	107 (34.6)	0.940
Dabigatran	120 (12.9)	81 (13.0)	39 (12.6)	0.939
Rivaroxaban	491 (52.8)	328 (52.8)	163 (52.8)	1.000
Triple antithrombotic therapy	504 (54.2)	365 (58.8)	139 (45.0)	<0.001
Statins	891 (95.8)	599 (96.5)	292 (94.5)	0.219
β blockers	788 (84.7)	521 (83.9)	267 (86.4)	0.365
ACEI/ARB	569 (61.2)	382 (61.5)	187 (60.5)	0.824
Spironolactone	273 (29.4)	134 (21.6)	139 (45.0)	<0.001
Diuretics	406 (43.7)	197 (31.7)	209 (67.6)	<0.001
Proton pump inhibitors	645 (69.4)	421 (67.8)	224 (72.5)	0.165

*AF, atrial fibrillation; ACS, acute coronary syndrome; PCI, percutaneous coronary intervention; ET-1, endothelin-1; ACEI, angiotensin-converting enzyme inhibitors; ARB, angiotensin II receptor blockers*.

### Factors Related to High Plasma Big ET-1 Levels

Multivariable logistic regression indicated that systolic blood pressure, type of AF, previous coronary stent implantation, white blood cell count, hemoglobin, red cell distribution width, platelet count, uric acid, free fatty acid, N-terminal pro-B type natriuretic peptide, and left ventricular ejection fraction were independently related to big ET-1 ≥ 0.54 pmol/l (all *p* < 0.05) (Table 2).

**Table 2:** Independent factors related to big ET-1 ≥ 0.54 mmol/l^*^.

**Variables**	**OR (95%CI)**	***p*-value**
Systolic blood pressure, per 10 mmHg	1.110 (1.009-1.220)	0.031
Type of AF, *n* (%)		0.020
Paroxysmal	1 (reference)	
Persistent	1.688 (1.117–2.552)	0.013
Permanent	1.554 (1.060–2.279)	0.024
Previous coronary stent implantation	1.432 (1.002–2.048)	0.049
White blood cell count, per 1*10^9^/l	1.081 (1.004–1.164)	0.040
Hemoglobin, per 10 g/l	0.807 (0.731–0.891)	<0.001
Red blood cell distribution width, per 1%	1.207 (1.041–1.399)	0.013
Platelet count, per 10*10^9^/l	0.939 (0.913–0.966)	<0.001
Uric acid, per 10 umol/l	1.022 (1.007–1.036)	0.003
Free fatty acid, per 0.1 mmol/l	1.072 (1.014–1.133)	0.015
N-terminal pro-B type natriuretic peptide, per 100pg/ml	1.016 (1.008–1.024)	<0.001
Left ventricular ejection fraction, per 10%	0.760 (0.640–0.901)	0.002

*OR, odds ratio; CI, confidence interval*.

* *Factors related to big ET-1 ≥ 0.54 mmol/l were identified by backward-stepwise logistic regression models including age, male gender, body mass index, heart rate, systolic blood pressure, type of AF, qualifying index event, smoking history, drinking history, hypertension, diabetes mellitus, previous myocardial infarction, previous coronary stent implantation, previous CABG, heart failure, previous stroke/transient ischemic attack, previous bleeding events, chronic obstructive pulmonary disease, peripheral arterial disease, white blood cell count, neutrophil-lymphocyte ratio, hemoglobin, red cell distribution widths, platelet count, creatinine clearance, uric acid, free fatty acid, low-density lipoprotein cholesterol, hemoglobin A1c, N-terminal pro-B type natriuretic peptide, high sensitivity C reactive protein, left atrial diameter, left ventricular end-diastolic diameter, left ventricular ejection fraction*.

### Follow-Up and Outcomes

**Figure 1:**
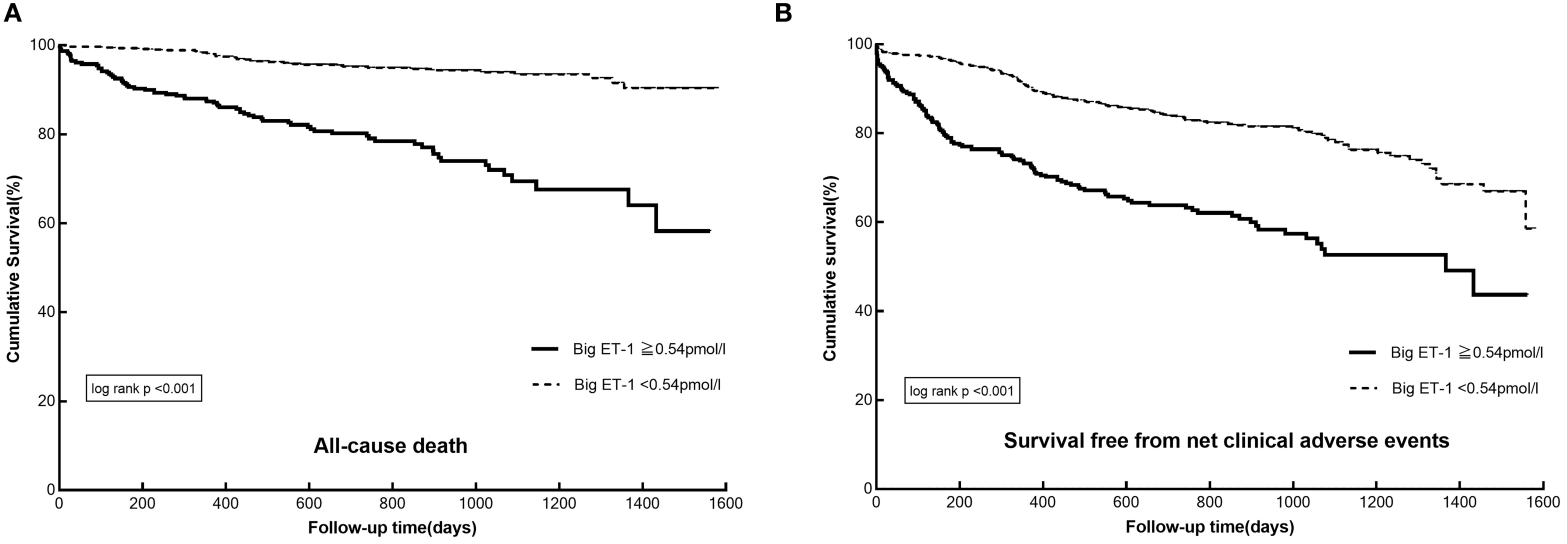
The Kaplan–Meier survival curves in patients with AF and ACS or undergoing PCI divided by plasma big ET-1 levels: **(A)** cumulative survival, **(B)** survival free from net adverse clinical events. AF, atrial fibrillation; ACS, acute coronary syndrome; PCI, percutaneous coronary interventions; big ET-1, big endothelin-1.

During a median follow-up of 2.3 years, 109 all-cause deaths (11.7%) and 88 cardiovascular deaths (9.5%) occurred. A total of 217 patients (23.3%) have experienced MACE, of whom 57 patients (6.1%) have developed stroke and 47 patients (5.1%) have suffered from myocardial infarction. As to the safety endpoints, 49 patients (5.3%) and 166 patients (17.8%) have experienced BARC class ≥ 3 and BARC class ≥ 2 bleeding, respectively. In patients with big ET-1 ≥ 0.54 pmol/l, the incidences of the primary (all-cause death: 23.6 vs. 5.8%, NACE: 39.5 vs. 20.1%) and secondary endpoints (cardiovascular death: 20.4 vs. 4.0%, MACE: 35.0 vs. 17.6%, BARC class ≥ 3 bleeding: 9.4 vs. 3.2%, BARC class ≥ 2 bleeding: 24.9 vs. 14.3%) were remarkably higher than those in patients with big ET-1 <0.54 pmol/l (all *p* < 0.05) (Table 3). The Kaplan-Meier survival curves presented significant differences between the two groups in the risk of all-cause death (log-rank *p* < 0.001) and NACE (log-rank *p* < 0.001) (Figure 1).

**Table 3:** Association between plasma big ET-1 levels and long-term outcomes in patients with AF and ACS or undergoing PCI.

**Endpoints, *n* (%)**	**Big ET-1 <0.54 pmol/l (*n* = 621)**	**Big ET-1 ≥0.54 pmol/l (*n* = 309)**	***p*-value**	**Univariable cox regression**		Multivariable cox regression^*^	
				**HR (95%CI)**	***p*-value**	**HR (95%CI)**	***p*-value**
**Primary endpoints**							
All-cause death	36 (5.8)	73 (23.6)	<0.001	5.07 (3.39–7.56)	<0.001	1.73 (1.10–2.71)	0.018
Net adverse clinical events	125 (20.1)	122 (39.5)	<0.001	2.58 (2.01–3.32)	<0.001	1.63 (1.23–2.16)	0.001
**Secondary endpoints**							
Cardiovascular death	25 (4.0)	63 (20.4)	<0.001	6.31 (3.96–10.04)	<0.001	1.72 (1.01–2.92)	0.046
Major adverse cardiovascular events	109 (17.6)	108 (35.0)	<0.001	2.54 (1.94–3.32)	<0.001	1.60 (1.19–2.16)	0.002
Stroke	35 (5.6)	22 (7.1)	0.457				
Non-CNS thromboembolism	7 (1.1)	3 (1.0)	1.000				
Target vessel revascularization	29 (4.7)	8 (2.6)	0.177				
Myocardial infarction	22 (3.5)	25 (8.1)	0.005				
Definite or probable stent thrombosis	2 (0.3)	2 (0.6)	0.604				
**Safety endpoints**							
BARC class ≥ 3 bleeding	20 (3.2)	29 (9.4)	<0.001	3.58 (2.02–6.34)	<0.001	2.21 (1.16–4.22)	0.016
BARC class ≥ 2 bleeding	89 (14.3)	77 (24.9)	<0.001	2.19 (1.61–2.97)	<0.001	1.91 (1.36–2.70)	<0.001
**Bleeding according to BARC criteria**							
BARC type 1 bleeding	143 (23.0)	61 (19.7)	0.291				
BARC type 2 bleeding	69 (11.1)	48 (15.5)	0.070				
BARC type 3a bleeding	5 (0.8)	16 (5.2)	<0.001				
BARC type 3b bleeding	3 (0.5)	7 (2.3)	0.019				
BARC type 3c bleeding	12 (1.9)	4 (1.3)	0.662				
BARC type 5 bleeding	0 (0.0)	2 (0.6)	0.110				

*ET-1, endothelin-1; AF, atrial fibrillation; ACS, acute coronary syndrome; PCI, percutaneous coronary intervention; CNS, central nervous system; BARC, bleeding academic research consortium; HR, hazard ratio; CI, confidence interval*.

* *Adjusted for age, male gender, body mass index, heart rate, systolic blood pressure, type of AF, qualifying index event, smoking history, drinking history, hypertension, diabetes mellitus, previous myocardial infarction, previous coronary stent implantation, previous CABG, heart failure, previous stroke/transient ischemic attack, previous bleeding events, chronic obstructive pulmonary disease, peripheral arterial disease, white blood cell count, neutrophil-lymphocyte ratio, hemoglobin, red cell distribution widths, platelet count, creatinine clearance, uric acid, free fatty acid, low-density lipoprotein cholesterol, hemoglobin A1c, N-terminal pro-B type natriuretic peptide, high sensitivity C reactive protein, left atrial diameter, left ventricular end-diastolic diameter, left ventricular ejection fraction, triple antithrombotic therapy, β blockers, ACEI/ARB, spironolactone, diuretics, and proton pump inhibitors by backward LR method*.

**Figure 2:**
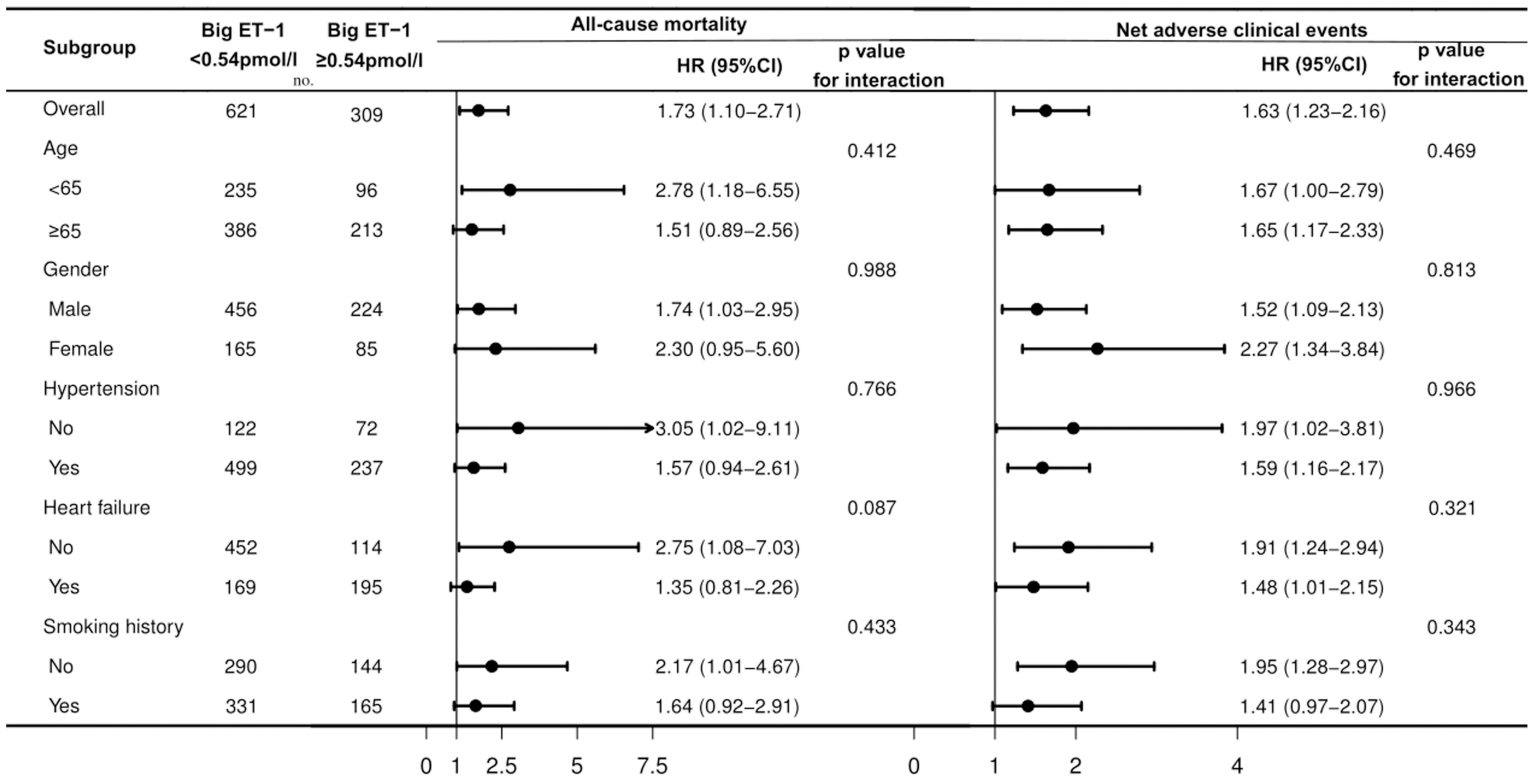
Subgroup analysis for associations between big ET-1 ≥ 0.54 pmol/l and the primary endpoints in patients with AF and ACS or undergoing PCI. AF, atrial fibrillation; ACS, acute coronary syndrome; PCI, percutaneous coronary interventions; big ET-1, big endothelin-1; HR, hazard ratio; CI, confidence interval.

Univariable Cox regressions demonstrated that the plasma big ET-1 level was significantly associated with incidences of the primary and secondary endpoints. After adjustment for potential confounders in multivariable Cox regressions, big ET-1 ≥ 0.54 pmol/l was indicated to be an independent predictor of all-cause death [HR (95%CI): 1.73 (1.10–2.71), *p* = 0.018] and NACE [HR (95%CI): 1.63 (1.23–2.16), *p* = 0.001]. Additionally, elevated plasma big ET-1 levels were also significantly associated with increased risk of cardiovascular death [HR (95%CI): 1.72 (1.01–2.92), *p* = 0.046], MACE [HR (95%CI): 1.60 (1.19–2.16), *p* = 0.002], BARC class ≥ 3 [HR (95%CI): 2.21 (1.16–4.22), *p* = 0.016], and BARC class ≥ 2 bleeding [HR (95%CI): 1.91 (1.36–2.70), *p* < 0.001] (shown in Table 3). The subgroup analyses for all-cause mortality and NACE demonstrated that no significant interaction existed between plasma big ET-1 levels and age, gender, hypertension, heart failure, and smoking history (all *p*-values for interaction > 0.05) (Figure 2).

## Discussion

In the present cohort, patients with high plasma big ET-1 levels tended to have more comorbidities, impaired cardiac function, elevated inflammatory levels, and worse prognosis. Factors reflecting activated inflammation and cardiac dysfunction were related to big ET-1 ≥ 0.54 pmol/l. In patients with AF and ACS or undergoing PCI, big ET-1 ≥ 0.54 pmol/l was an independent predictor of all-cause death, NACE, cardiovascular death, MACE, BARC class ≥ 3 bleeding, and BARC class ≥ 2 bleeding. The results of subgroup analysis were consistent with the overall results for these patients.

Risk stratification based on clinical and laboratory variables is essential in prognosis evaluation and management guidance for patients with AF and ACS or undergoing PCI (1, 2).

In the present study, patients in the high big ET-1 group tended to have more comorbidities, impaired cardiac function, and elevated inflammatory levels. Factors reflecting inflammation (white blood cell count, red cell distribution width, platelet count, free fatty acid) and cardiac function (N-terminal pro-B type natriuretic peptide, left ventricular ejection fraction) were detected to be independently associated with plasma big ET-1 levels, which were consistent with previous reports (17). This relationship indicated that big ET-1 ≥ 0.54 pmol/l might be a useful marker of activated inflammation state and impaired cardiac function. Previous researches have revealed that ET-1 plays a crucial role in endothelial dysfunction, inflammation, and myocardial remodeling (7, 8, 22). Accordingly, big ET-1 might contribute to the occurrence and progression of AF and CAD through facilitating these processes.

Plasma concentrations of ET-1 and big ET-1 have been detected to be related to the prognosis of abundant cardiovascular diseases (12–15, 17, 18). Yip et al. (23) found that ET-1 was a strong predictor of 30-day mortality and major adverse cardiovascular events in ST segment-elevation myocardial infarction patients undergoing primary PCI. A cohort of 110 first-acute myocardial infarction patients undergoing primary PCI showed that higher ET-1 level was related to increased incidences of cardiogenic shock and cardiac death (24). Several studies have also detected a significant correlation between ET-1 and long-term mortality in patients with acute myocardial infarction (13, 16). As to stable CAD, big ET-1 was also an independent predictor of 2-year cardiovascular outcomes (15). Another study including 6,150 patients with three-vessel disease indicated that big ET-1 was independently associated with long-term mortality (14). Wu et al. (17) enrolled 716 patients with AF and found that the elevated big ET-1 level was an independent predictor of long-term mortality and major adverse events during a median follow-up of 3 years. Additionally, the correlations between plasma big ET-1 levels and prognosis of other cardiovascular diseases [such as heart failure (12), hypertrophic cardiomyopathy (18)] have also been identified by abundant studies.

To the best of our knowledge, our study indicated that elevated plasma big ET-1 levels were associated with an increased risk of adverse outcomes in patients with AF and ACS or undergoing PCI for the first time. Potential mechanisms for the association between big ET-1 and prognosis in these patients have not been fully elucidated and might be manifold. Firstly, elevated levels of ET-1 and big ET-1 are practical markers for the activation of inflammation (7, 8, 22). Besides endothelial cells, inflammatory cells such as polymorphonuclear leukocytes and macrophages are also important sources of ET-1 (25, 26). It's well-established that inflammation plays an important part in the initiation and progression of AF and CAD (27). ET-1 could induce the adherence of neutrophils to vascular endothelial and myocardium cells by upregulating adhesive molecule expression (28). Meanwhile, elevated plasma ET-1 levels are related to the enhancement of oxidative stress and activation of various inflammatory factors in the inflammation cascade. All of these have been indicated to participate in the formation and aggravation of artery atherosclerotic and myocardial fibrosis, which might contribute to the unsatisfactory prognosis of patients with AF and CAD (29, 30). Secondly, ET-1 is closely related to endothelial dysfunction (31, 32). ET-1 is a potent vasoconstrictor (32) and is associated with decreased synthesis and increased degradation of nitric oxide (7, 8, 22). Accordingly, ET-1 plays an important role in maintaining a balance between vasoconstriction and vasodilatation (32). Abundant studies have demonstrated that endothelial dysfunction participated in the development of CAD and AF (23, 33, 34). Thirdly, ET-1 is an autocrine and paracrine mediator with mitogenic and inotropic effects in the myocardium. Previous researches have detected the relationship of ET-1 with vascular and myocardial remodeling (9–11). Several studies demonstrated that ET-1 was related to the modulation of post-infarct left ventricular remodeling (11) and reduced left ventricular ejection fraction (13). On the other hand, Mayyas et al. found that ET-1 was positively associated with left atrial size, heart failure, AF persistence, and severity of mitral regurgitation (9). Structural remodeling and electrical remodeling have been verified to be essential in the occurrence, maintenance, recurrence, and progression of AF (35). ET-1 has been indicated to carry arrhythmogenic effects (36) and play an essential role in the initiation and perpetuation of AF (37). In patients with AF and ACS or undergoing PCI, elevated plasma ET-1 levels might contribute to a worse prognosis through facilitating atrial and ventricular remodeling (9–11). Finally, ET-1 has been indicated to be associated with coronary artery calcification, reperfusion injury, no-reflow, and stent thrombosis (7, 8, 13, 38, 39). Additionally, previous researches have detected a relation of ET-1 with promoted platelet aggregation and activated prothrombotic state (7, 40). All of these might contribute to increased thromboembolic risk and unfavored prognosis. In this study, subgroup analysis indicated a consistent relationship between plasma big ET-1 levels and all-cause mortality in patients with AF and ACS or undergoing PCI. The positive correlation between elevated big ET-1 and prognosis might be of great value in clinical routine practice. The plasma big ET-1 level could act as a useful biomarker for risk stratification, prognosis evaluation, and management guidance in patients with AF and ACS or undergoing PCI. Identification of high-risk patients and adoption of intensified management strategies might do a favor in improving these patient's prognosis (1, 2).

Several limitations should be mentioned in this study. First, due to inherent defects of observational studies, a definite causal association between big ET-1 and clinical endpoints could not be inferred. On the other hand, although multivariable regression analysis has been performed to adjust for potential confounders, the list of relevant variables for adverse outcomes could hardly be exhaustive. The results should be interpreted cautiously. Second, this study only tested plasma big ET-1 levels at baseline and lacked serial data of plasma big ET-1 levels during the follow-up. Dynamic monitoring of plasma big ET-1 levels might provide more information for prognosis prediction. In the present study, big ET-1 rather than ET-1 levels, plasma rather than cardiac levels were measured. These might influence the accuracy of our results. The exact association between ET-1 and prognosis in patients with AF and ACS or undergoing PCI might be explored in the future. Third, this study was conducted in a single center in China. The homogeneity of participants and management strategies might limit the generalizability of our conclusions to other populations. Finally, the sample size was relatively small in this study, which might limit the statistical power. Further well-designed prospective studies with a larger sample size would provide more evidence for this issue.

## Conclusion

In patients with AF and ACS or undergoing PCI, the elevated big ET-1 level was an independent predictor of all-cause death, NACE, cardiovascular death, MACE, BARC class ≥ 3 bleeding, and BARC class ≥ 2 bleeding.

## Data Availability Statement

The raw data supporting the conclusions of this article will be made available by the authors, without undue reservation.

## Ethics Statement

The studies involving human participants were reviewed and approved by the Ethics Committee of Fuwai Hospital. The patients/participants provided their written informed consent to participate in this study.

## Author Contributions

S-qL: collected the data, performed the statistical analysis, drafted, and wrote the manuscript. Y-mY and JZ: designed and revised the manuscript. SW, JW, HZ, and X-hS: collected the data. All authors read and approved the final manuscript.

## Funding

This work was supported by Capital's Funds for Health Improvement and Research (No. 2018-2-4031) and Capital‘s Funds for Research and Application of Clinical Diagnosis and Treatment Technology (Z191100006619121).

## Conflict of Interest

The authors declare that the research was conducted in the absence of any commercial or financial relationships that could be construed as a potential conflict of interest.

## Publisher's Note

All claims expressed in this article are solely those of the authors and do not necessarily represent those of their affiliated organizations, or those of the publisher, the editors and the reviewers. Any product that may be evaluated in this article, or claim that may be made by its manufacturer, is not guaranteed or endorsed by the publisher.

## Supplementary Material

Supplementary Figure 1 Flowchart for subject selection. AF, atrial fibrillation; CAD, coronary artery disease; ACS, acute coronary syndrome; PCI, percutaneous coronary interventions; big ET-1, big endothelin-1.
