# Supplementary figures and images for: Plasma Big Endothelin-1 Levels and Long-Term Outcomes in Patients With Atrial Fibrillation and Acute Coronary Syndrome or Undergoing Percutaneous Coronary Intervention
Source: Front Cardiovasc Med. 2022 Mar 3;9:756082. doi: 10.3389/fcvm.2022.756082 (PMC8927675; doi:10.3389/fcvm.2022.756082)

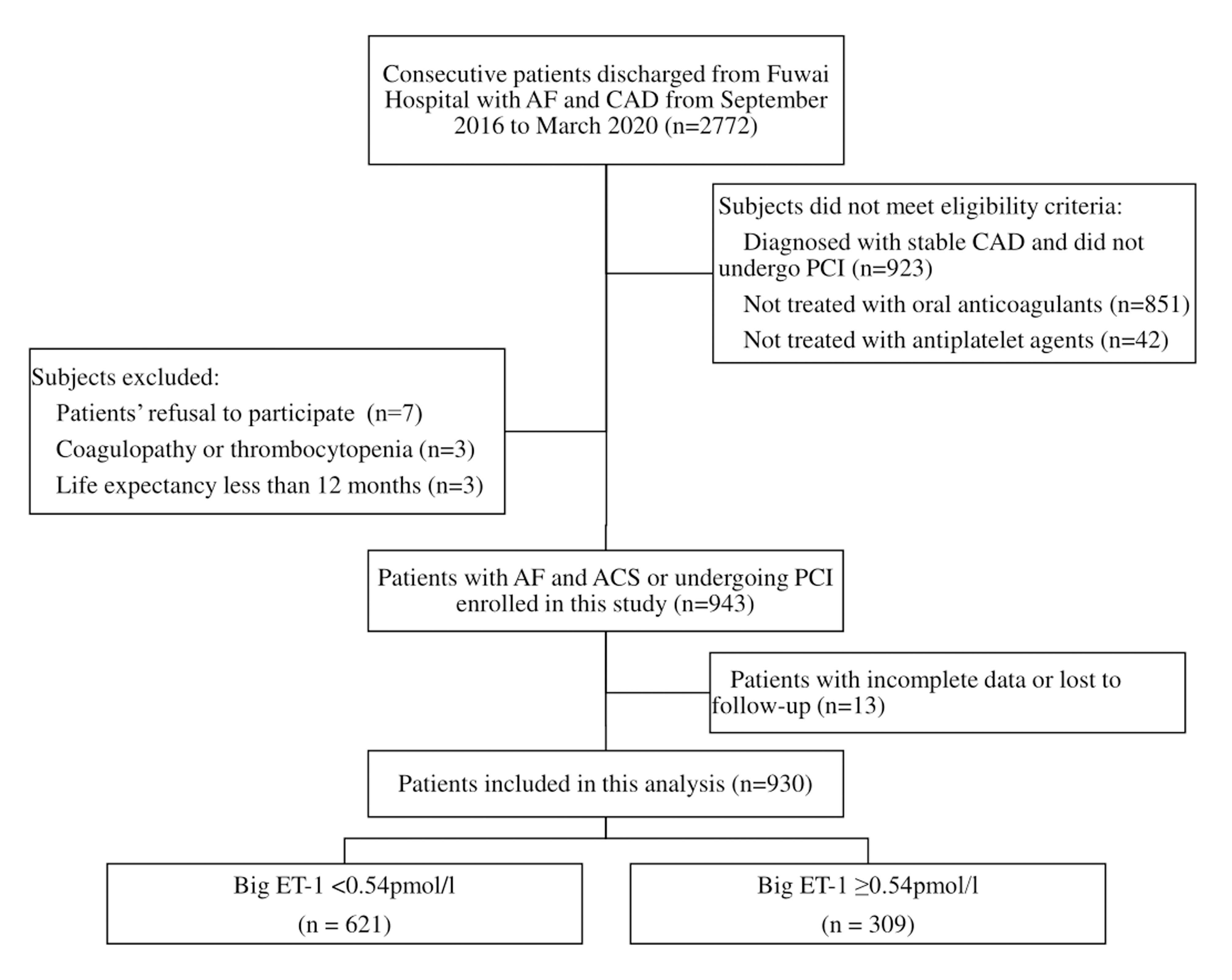

Supplement: Supplementary Figure 1 — Flowchart for subject selection. AF, atrial fibrillation; CAD, coronary artery disease; ACS, acute coronary syndrome; PCI, percutaneous coronary interventions; big ET-1, big endothelin-1. [file Image_1.TIF]
